# Deep Learning‐Based MRI Volumetry for Living Kidney Donor Assessment: A New Tool for Predicting Post‐Donation Renal Function

**DOI:** 10.1111/ctr.70338

**Published:** 2025-10-07

**Authors:** Dominik Thomas Koch, Felix Oliver Hofmann, Dimitrios Trompoukis, Malte Schirren, Severin Jacobi, Tobias Seibt, Stephan Kemmner, Maximilian Scheifele, Matthias Ilmer, Bernhard Renz, Jens Werner, Manfred Stangl, Markus Guba, Dionysios Koliogiannis

**Affiliations:** ^1^ Department of General, Visceral and Transplantation Surgery LMU University Hospital LMU Munich Munich Germany; ^2^ Transplant Center LMU University Hospital LMU Munich Munich Germany; ^3^ Department of Nuclear Medicine LMU University Hospital LMU Munich Munich Germany; ^4^ Division of General Surgery Mayo Clinic Rochester Minnesota USA

**Keywords:** diagnostic techniques and imaging, deep learning‐based MRI volumetry, kidney function, kidney transplantation, living kidney donation, magnetic resonance imaging (MRI), renal scintigraphy

## Abstract

**Background:**

Living kidney donation is a crucial option for addressing the global organ shortage and providing kidney transplantation for patients suffering from end‐stage kidney disease. Ensuring donor safety necessitates a comprehensive preoperative assessment of kidney anatomy and function. This study evaluates the relationship between kidney volumes derived from deep learning‐based MRI volumetry, intraoperative kidney volume measurements, split renal function measured by renal scintigraphy, and post‐donation eGFR. Deep learning‐based MRI volumetry is hypothesized to be a reliable method with good correlation.

**Methods:**

This retrospective study analyzed 178 living kidney donors. Deep learning MRI volumetry‐based kidney volumes were compared with intraoperative volumes of the explanted donor kidneys obtained using the water displacement method. Additionally, MRI‐based volume ratios were compared with scintigraphy‐based split renal function ratios to determine their ability to predict the kidney with poorer renal function and post‐donation eGFR.

**Results:**

Deep learning‐based MRI volumetry strongly correlated with intraoperatively measured kidney volumes (Pearson's correlation; *r* = 0.7671; *p* < 0.0001), confirming its precision in volume estimation. Although MRI‐based kidney volume ratios demonstrated only a moderate correlation with scintigraphy‐based split renal function ratios (*r* = 0.4798), MRI volumetry correlated with 1‐year post‐donation eGFR. It tended to be better than renal scintigraphy (*r* = 0.6829 versus *r* = 0.6191).

**Conclusion:**

Deep learning‐based MRI volumetry is a reliable, non‐invasive tool for estimating kidney volumes in living donors, offering a radiation‐free alternative for preoperative assessment. While it differs from renal scintigraphy in evaluating split renal function ratios, its correlation with post‐donation eGFR tends to be better, supporting its potential role in living kidney donor assessment.

**Abbreviations**
CKD‐EPI: Chronic Kidney Disease Epidemiology Collaboration
eGFR: estimated glomerular filtration rate
HTK: histidine‐tryptophan‐ketoglutarate
MAG3: mercaptoacetyltriglycine
MRI: magnetic resonance imaging
Scr: serum creatinine
SD: standard deviation
SRF: split renal function
TER: tubular extraction rate

## 1 Introduction

Kidney transplantation is a life‐changing procedure for patients with end‐stage kidney disease, eliminating the need for dialysis, significantly improving survival, and enhancing quality of life [1, 2, 3, 4, 5, 6]. However, the ongoing shortage of deceased donors remains a major challenge, emphasizing the essential role of living kidney donation in addressing this gap [7, 8]. Compared to deceased donor transplants, living donor kidney transplantation offers superior graft survival and long‐term outcomes [9, 10]. Nevertheless, ensuring the safety and well‐being of living kidney donors is paramount, requiring a meticulous preoperative assessment of kidney anatomy and function.

Preoperative evaluation for living kidney donors traditionally includes multiple tests, with renal scintigraphy used to assess split renal function (SRF) and magnetic resonance imaging (MRI) for anatomical evaluation. While previous studies have suggested a correlation between kidney volume and function [11, 12, 13], this relationship remains controversial. Although larger kidneys generally exhibit greater functional capacity, nephron density, vascular supply, and fibrosis make this association more complex. Even more, in clinical practice, manual segmentation of kidney volumes ‐ meaning the process of separating and labeling the kidneys within CT or MRI scans – is resource‐intensive and time‐consuming, limiting its routine application.

Advances in artificial intelligence, particularly deep learning‐based models, have made automated segmentations feasible, fast, and readily available. This study aims to evaluate the accuracy of deep learning‐based MRI volumetry in predicting kidney volumes by comparing MRI‐based volumes with intraoperatively measured volumes. Additionally, it investigates the correlation between MRI‐based volumetry, renal scintigraphy‐based SRF, and post‐donation eGFR of the kidney remaining in the donor to assess its potential as a reliable tool in preoperative donor evaluation.

## 2 Materials and Methods

### 2.1 Study Design and Study Population

This retrospective, single‐center study analyzed 178 living kidney donors who underwent preoperative evaluation and kidney donation at the Department of General, Visceral and Transplantation Surgery, LMU University Hospital, LMU Munich, Germany, between 2016 and 2024. Inclusion criteria required a complete preoperative MRI of both kidneys and at least one reference measure, such as preoperative renal scintigraphy, intraoperative kidney volume assessment, or serum creatinine follow‐up data. Donors with incomplete MRI scans or missing reference data were excluded.

Existing MRI scans were used for deep learning‐based kidney segmentation and volumetry. The MRI‐based kidney volumes were compared with intraoperatively measured volumes obtained through the water displacement method. The primary objective was to determine the correlation between MRI‐based volumetry and intraoperative measurements, as well as to evaluate its potential for predicting residual kidney volume and function in the donors.

### 2.2 Data Sources

All data used in this study were pre‐existing, and no additional questionnaires, tests, or procedures were required. As a result, study‐specific informed consent was not required. Data collection was conducted by authors adhering to data protection regulations, and all analyses were performed following irreversible anonymization of the data.

Donor data, including graft volume and partial renal function determined by scintigraphy, were extracted from digitized institutional medical records. Preoperative MRI datasets were reviewed, and the T1‐VIBE axial images were exported with a 512 × 512 pixel resolution. Donors were excluded if this imaging phase was incomplete or unavailable.

### 2.3 Transplantation Program

Our institution's living donor kidney transplantation program has been established for many years and is highly standardized. Only candidates with a projected pre‐donation lifetime risk of less than 1% for end‐stage kidney disease, calculated by the parameters age, gender, race, eGFR, systolic blood pressure, presence of hypertension medication, BMI, presence of diabetes, urine albumin to creatinine ratio, and smoking history, can be further evaluated for living kidney donation. Once deemed eligible, donors provide informed consent, and further evaluations are carried out.

Pre‐transplantation assessments routinely include an MRI with an intravenously applied contrast agent performed to evaluate the donor's anatomy, especially to identify additional or aberrant vessels. A renal scintigraphy is routinely performed to determine split renal function.

After obtaining all examinations, cases are individually reviewed by our kidney transplantation board, consisting of nephrologists, transplant surgeons, psychiatrists, and independent members. If transplantation is approved, the procedure is scheduled, ensuring sufficient time for donor and recipient consideration. The explanted kidney's volume is measured using the water displacement method during the operation.

### 2.4 MRI Segmentation

For kidney segmentation, meaning the process of separating and labeling the kidneys within the MRI scans, Digital Imaging and Communications in Medicine (DICOM) images were exported and converted to NIfTI format. Then, kidney segmentation was performed using the MRSegmentator package (v1.1, available from https://github.com/hhaentze/MRSegmentator), a deep learning model designed for segmenting 40 anatomical structures in MRI scans [14]. The tool uses the nnU‐Net framework [15], enabling self‐configuring deep learning‐based biomedical image segmentation.

**FIGURE 1:**
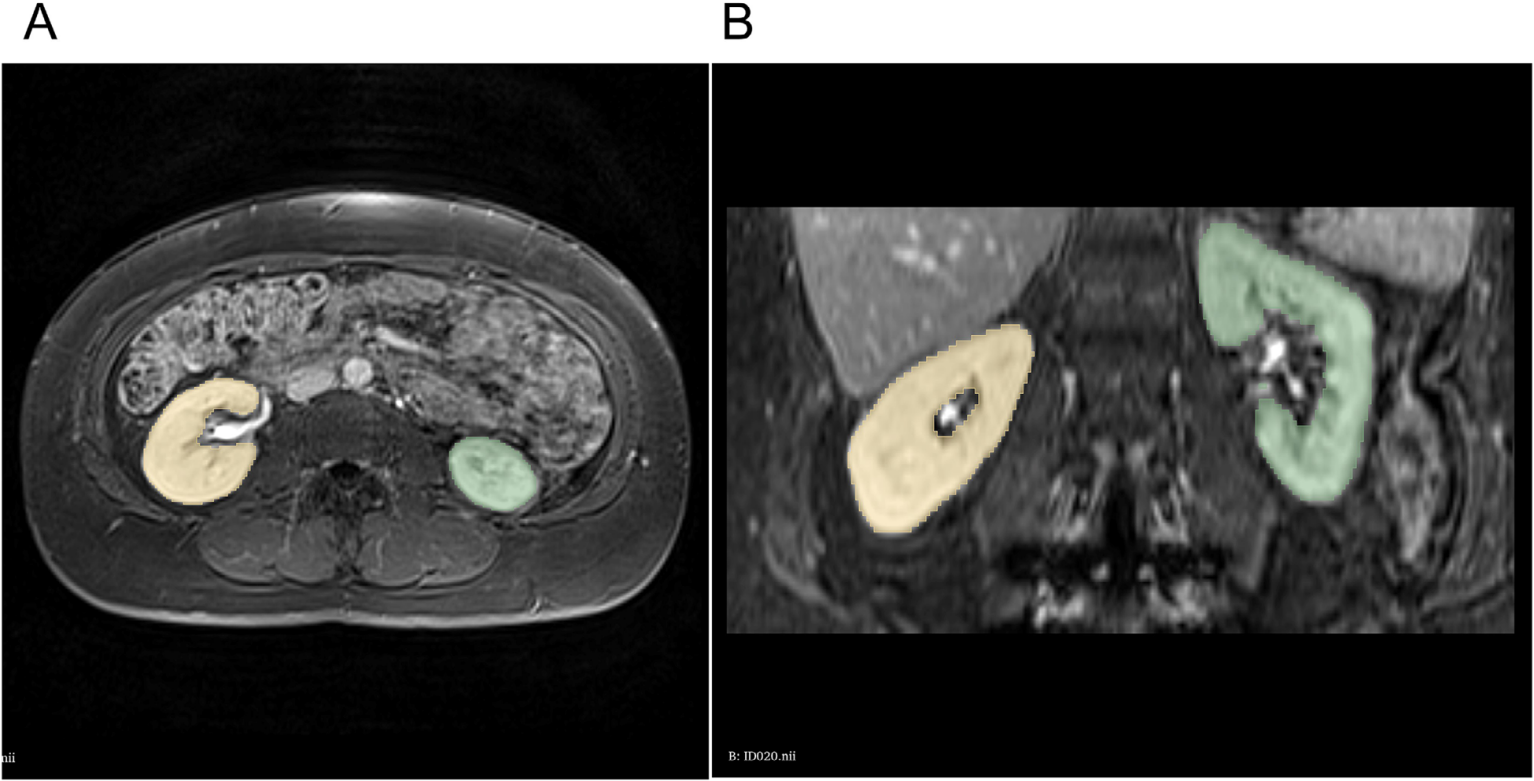
MRI T1‐VIBE axial (A) and coronal (B) images with labeled left (green) and right (yellow) kidney.

All generated segmentation labels were manually reviewed and adjusted as needed using the 3D Slicer software [16]. The labels encompassed the entire kidney, including the renal cortex and medulla, while excluding the renal pelvis and perirenal adipose tissue. Any visible cysts were included in the segmentation (Figure 1). No tumors were expected or observed in donor kidneys.

Kidney volumes were computed by multiplying the voxel volume by the total number of voxels assigned to each segmented kidney. Intensity‐adjusted volumes were determined by multiplying the computed kidney volumes by the mean intensity values of all included voxels.

### 2.5 Assessment of Split Renal Function via Renal Scintigraphy

Determination of the split renal function (SRF) was part of the standard preoperative assessment of living kidney donors using a standard Tc99m‐mercaptoacetyltriglycine (MAG3) scintigraphy protocol. The potential donors received 1000 mL of 0.9% sodium chloride intravenously for proper hydration before tracer administration at a standard 100 MBq Tc‐99m MAG3 dose. Dynamic imaging was performed over 30 min with 20 mg furosemide administration at 15 min post tracer injection, followed by a static post‐micturition image after voiding. SRF was calculated using the integral method by measuring renal activity during the 1–3‐min post‐injection period while including a background correction. Tubular extraction rate (TER) was calculated using Bubeck's approach with a blood sample at minute 20 and minute 28.

### 2.6 Intraoperative Kidney Volume Measurement

Donor kidneys were cooled and flushed with histidine‐tryptophan‐ketoglutarate (HTK) containing heparin immediately after explantation. The explanted kidney's volume was then measured using the “water displacement” method. A graduated container was filled with sterile isotonic saline solution to the top, and the kidneys were then submerged completely in the fluid. The assessed fluid level difference after taking out the kidney represented the volume of the kidney.

### 2.7 Calculation of the Estimated Glomerular Filtration Rate

The estimated glomerular filtration rate (eGFR) was calculated using the 2021 Chronic Kidney Disease Epidemiology Collaboration (CKD‐EPI) creatinine equation refit without the race variable [17]:

$$\begin{array}{ccc} eGFR & & =142\;\times \min{\left(\frac{Scr}{\kappa };1\right)}^{\alpha }\times \max{\left(\frac{Scr}{\kappa };1\right)}^{-1.200}\\ & & \times \;0.9938^{age}\times \;1.012\;\left[if\;female\right] \end{array}$$

Scr, serum creatinine [mg/dl]; κ, 0.7 (females) or 0.9 (males); α, −0.241 (females) or −0.302 (males); min, indicates the minimum of $\frac{Scr}{\kappa }$ or 1; max, indicates the maximum of $\frac{Scr}{\kappa }$ or 1.

### 2.8 Statistical Analysis

Statistical analysis and chart generation were performed using GraphPad Prism 10 (GraphPad Software, San Diego, USA). The correlation between the MRI‐based kidney volume measurements and intraoperatively measured volumes of the transplanted organ was analyzed using Pearson's correlation coefficient. Bland–Altman analysis was conducted to assess the respective agreement. Likewise, the relationship between the MRI‐based kidney volume ratio and the renal scintigraphy‐based SRF ratio, as well as the relationship with the post‐donation eGFR of both methods, was analyzed using Pearson's correlation coefficient. All tests were conducted two‐sided, with a significance level of 0.05.

## 3 Results

A total of 178 living kidney donors with complete preoperative MRI scans of both donor kidneys and available reference data were included in the analysis. Intraoperative volume measurements were available for 162 of the 178 donors, renal scintigraphy data were available for all 178 donors, and 1‐year post‐donation eGFR data were available for 166 of the 178 donors.

### 3.1 Correlation Between Preoperative MRI‐Based Volumetry and Intraoperative Kidney Volume Measurements

For 162 living kidney donors, both complete preoperative MRI scans and intraoperative kidney volume measurements were available. MRI volumetry estimated a mean kidney volume of 154.2 mL (SD 29.52 mL) of the explanted kidney, ranging from 94.45 to 255.2 mL. Intraoperative measurements yielded larger volumes, with a mean of 180.5 mL (SD 44.53 mL) and a range of 100.0 to 310.0 mL.

**FIGURE 2:**
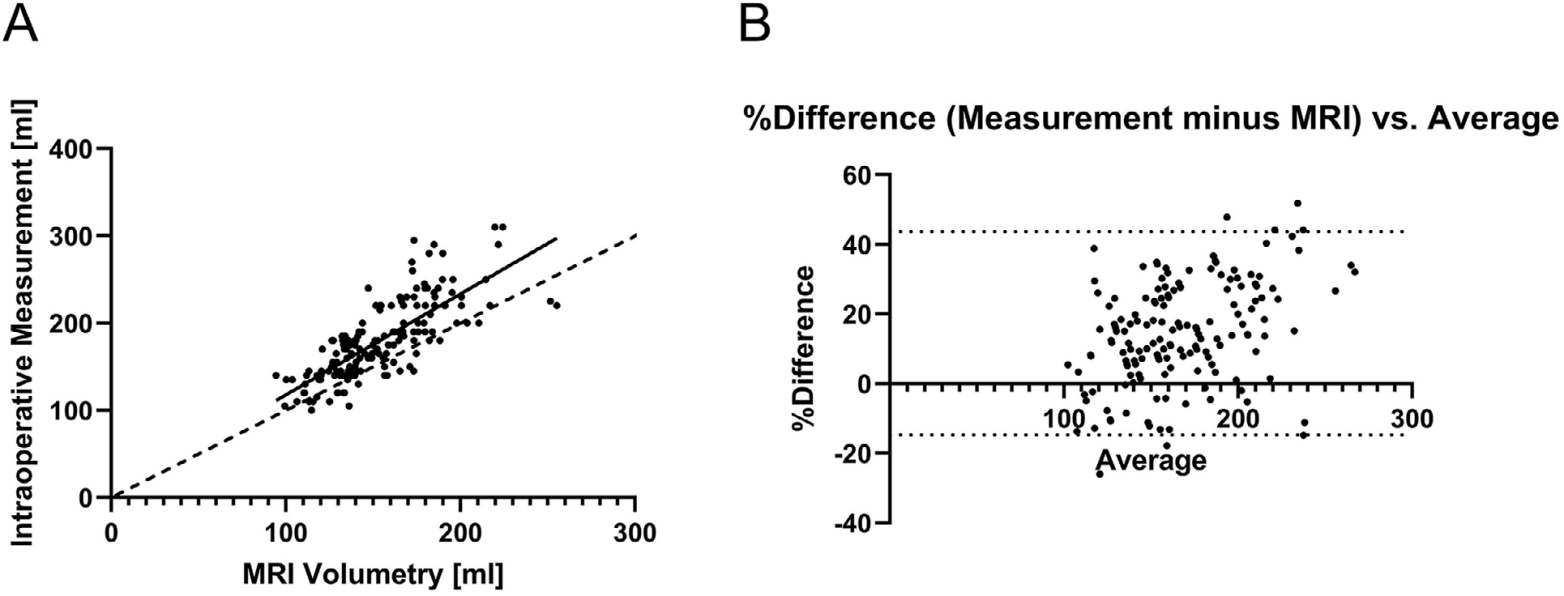
(A) Correlation between preoperative MRI‐based kidney volumes and intraoperatively measured volumes in 162 donors (*r* = 0.7671; *p* < 0.0001); (B) Bland–Altman plot comparing preoperative MRI‐based kidney volumes and intraoperatively measured volumes in 162 donors; The difference between the intraoperatively measured kidney volume and the MRI‐based volume (intraoperatively measured volume minus MRI‐based volume) as a percentage share of the mean of the two values is shown versus the mean.

For the validation of deep learning‐based MRI volumetry, the MRI volumetry‐estimated kidney volumes were correlated with intraoperative measurements. A strong and significant correlation was observed between MRI‐based kidney volumes and intraoperatively measured volumes, with a Pearson's correlation coefficient of *r* = 0.7671 (*p* < 0.0001; Figure 2A).

### 3.2 Bland–Altman Agreement Between MRI‐Based Volumetry and Intraoperative Kidney Volume Measurements

For the 162 living kidney donors, a Bland–Altman analysis was performed. Figure 2B shows the difference between the intraoperatively measured kidney volume and the MRI‐based volume (intraoperatively measured volume minus MRI‐based volume) as a percentage share of the mean of the two values versus the mean. The analysis revealed a mean bias of 14.50 mL (SD 14.90 mL) and 95% limits of agreement from −14.71  to 43.71 mL, indicating a systematic underestimation of kidney volumes by MRI volumetry.

### 3.3 Correlation Between MRI‐Based Kidney Volume Ratios and Renal Scintigraphy‐Based Split Renal Function Ratios

**FIGURE 3:**
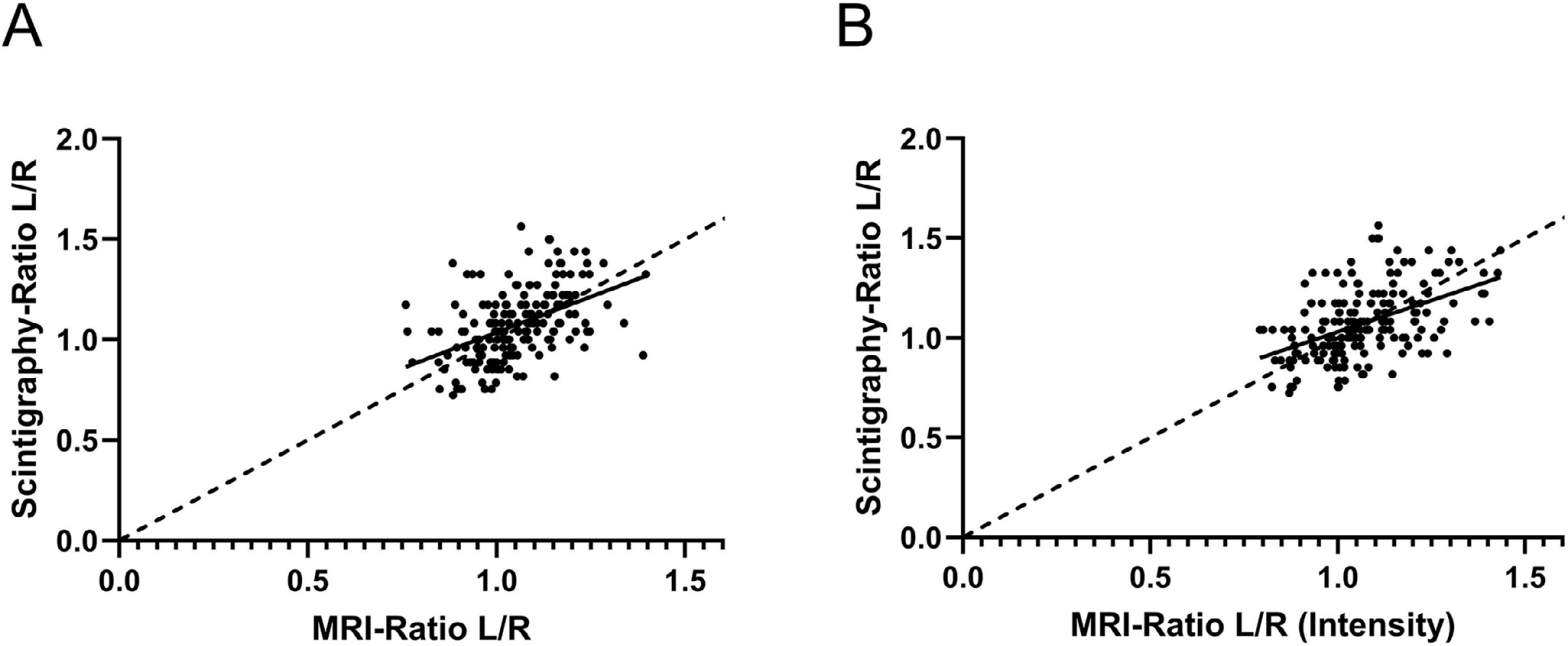
(A) Correlation between MRI‐based kidney volume ratios and renal scintigraphy‐based SRF ratios in 178 donors (*r* = 0.4581; *p* < 0.0001); (B) Correlation between intensity‐weighted MRI‐based kidney volume ratios and renal scintigraphy‐based SRF ratios in 178 donors (*r* = 0.4798; *p* < 0.0001).

Renal scintigraphy data were available for all 178 donors. The ratio of the left‐to‐right kidney volume, as determined by MRI volumetry, was compared to the renal scintigraphy‐based SRF ratio (Figure 3A). The correlation analysis revealed a Pearson's correlation coefficient of *r* = 0.4581 (*p* < 0.0001), indicating a moderate association between MRI‐based volume ratios and renal scintigraphy‐based SRF ratios.

The left‐to‐right ratio was also calculated for intensity‐corrected MRI‐based volumes (Figure 3B). This intensity‐weighted ratio showed a slightly improved correlation with the renal scintigraphy‐based ratio, with a Pearson's correlation coefficient of *r* = 0.4798 (*p* < 0.0001).

### 3.4 Comparison of MRI‐Based Side‐Selection and Standard Side‐Selection Based on Renal Scintigraphy

During the evaluation of living kidney donors, SRF is typically assessed using renal scintigraphy, with the left‐to‐right function ratio (L/R) serving as a key parameter. Without anatomical variations or other contraindications, the kidney with lower function is generally selected for donation to preserve the better‐functioning kidney in the donor.

A scintigraphy‐based ratio (L/R) of less than 1 suggests donation of the left kidney, while a ratio greater than 1 suggests donation of the right kidney. If the ratio equals 1, no scintigraphy‐based functional difference is observed between the kidneys, and no preference can be determined based on scintigraphy alone.

In this study, both scintigraphy‐based ratios and MRI‐based kidney volume ratios were available for all 178 donors. Among them, 17 donors had a scintigraphy‐based ratio of exactly 1, meaning no side preference could be established. For the remaining 161 donors, a scintigraphy‐based side suggestion was available and was compared with MRI‐based volumetric predictions. The two methods yielded consistent side recommendations in 115 (71.42%) cases.

### 3.5 Correlation Between Deep Learning‐Based MRI Volumetry and Post‐Donation eGFR

**FIGURE 4:**
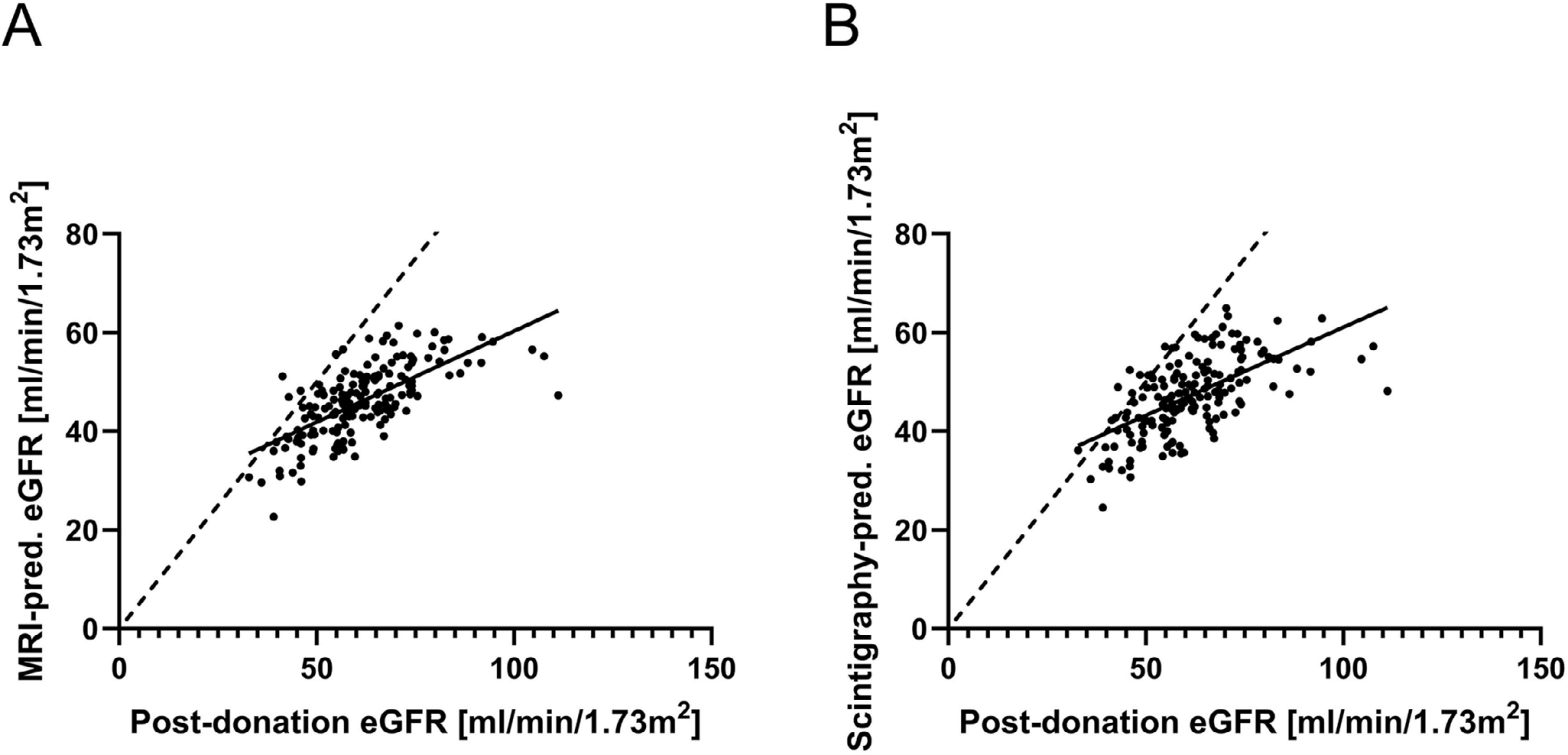
Correlation between predicted 1‐year post‐donation eGFR via MRI volumetry (A), respectively via renal scintigraphy (B) and actual 1‐year post‐donation eGFR assessed by serum creatinine levels.

To predict 1‐year post‐donation eGFR of the kidney remaining in the donor, pre‐donation eGFR was adjusted by the percentage of remaining renal volume as determined by MRI volumetry or adjusted by the percentage of remaining renal function as assessed by renal scintigraphy. The predicted values were compared with the actual 1‐year post‐donation eGFR, assessed by serum creatinine levels (Figure 4).

One‐year post‐donation eGFR data were available for 166 donors. The correlation between MRI volumetry‐based predictions and actual post‐donation eGFR was *r* = 0.6829 (*p* < 0.0001), while the correlation for renal scintigraphy‐based predictions was *r* = 0.6191 (*p* < 0.0001).

## 4 Discussion

This study yielded compelling findings, indicating a strong and significant correlation between preoperative deep learning‐based MRI volumetry and intraoperative kidney volume measurements, reinforcing the accuracy of MRI volumetry as a non‐invasive method for preoperative donor assessment. Our findings suggest that deep learning‐based MRI volumetry is not only a precise tool for estimating the volume of the donated kidney but also has the potential for predicting the volume and function of the kidney remaining in the donor with confidence.

A slight underestimation of kidney volume by MRI was observed, likely due to the exclusion of the renal pelvis during segmentation. In contrast, intraoperative volume measurements using the water displacement method include both the renal pelvis and the ureter. Moreover, residual adherent fat is measured with the water displacement method. The renal pelvis was deliberately excluded from MRI volumetry as it contains no functional renal tissue and is partially filled with fluid in situ, which may lead to overestimation if included.

Previous studies have shown that CT and MRI volumetry can predict not only kidney volumes but also donor renal function with reasonable accuracy. A systematic review by López‐Abad et al. analyzed 21 studies on MRI or CT volumetry [18], concluding that despite study heterogeneity, volumetry appears promising compared to scintigraphy, with favorable correlations and agreement. While 18 of these studies assessed CT volumetry [19, 20, 21, 22, 23, 24, 25, 26, 27, 28, 29, 30, 31, 32, 33, 34, 35, 36], only three evaluated MRI volumetry [11, 12, 13], with just two directly comparing MRI volumetry to renal scintigraphy. This highlights the limited body of research on MRI‐based volumetry, underscoring the need for further validation.

CT volumetry has demonstrated correlation coefficients with renal scintigraphy ranging from 0.21 [36] to 0.949 [27], while the two MRI volumetry studies have reported correlation coefficients of *r* = 0.58 [13] and *r* = 0.84 [11]. However, CT volumetry involves radiation exposure, which potentially increases lifetime cancer risks [37]. This makes MRI volumetry the preferable alternative, particularly for healthy living donors. MRI volumetry could serve as a viable method for estimating SRF while avoiding the risks associated with ionizing radiation.

Our study found only a moderate correlation between the MRI‐based kidney volume ratios and the scintigraphy‐based SRF ratios. Additionally, when renal scintigraphy identified functional asymmetry between the left and right kidneys, the MRI volumetry‐based side selection aligned with scintigraphy in only 71.42% of cases. These findings indicate that kidney volume alone does not fully correlate with conventional renal scintigraphy.

However, MRI volumetry demonstrated a strong correlation with post‐donation renal function. The correlation coefficient between MRI volumetry‐based predictions and 1‐year post‐donation eGFR was *r* = 0.6829, compared to *r* = 0.6191 for renal scintigraphy. This suggests that, while MRI‐based kidney volume ratios and SRF ratios do not correlate perfectly, MRI volumetry is associated with post‐donation eGFR and tends to correlate better than renal scintigraphy. Further prospective studies are needed to confirm this observation, and it needs to be emphasized that kidney volumes by any method are still only surrogates for the actual measured eGFR. Nevertheless, MRI volumetry may represent a complementary or even superior approach for determining the kidney most suitable for donation. Its utility becomes particularly evident when scintigraphy‐based SRF assessments reveal only minor lateral differences, and when anatomical variations ‐ such as the presence of multiple renal arteries, which may increase surgical risks ‐ render the decision less straightforward. In these contexts, MRI volumetry contributes to a more comprehensive risk‐benefit evaluation, thereby facilitating the decision‐making process.

## 5 Conclusion

This study highlights the potential of deep learning‐based MRI volumetry as a valuable tool for preoperative assessment in living kidney donors. Our findings demonstrate that MRI volumetry is highly reliable for estimating kidney volumes. Although MRI volumetry does not fully align with renal scintigraphy in assessing split renal function, its correlation with post‐donation eGFR even tends to be better.

Incorporating MRI volumetry into donor assessment could enhance preoperative planning by providing a non‐invasive, radiation‐free alternative for predicting residual kidney volume. Given the growing role of deep learning‐driven imaging analysis, MRI volumetry could be particularly beneficial when functional differences between kidneys are minimal.

Future research should focus on validating MRI volumetry in larger, multi‐center studies and evaluating its cost‐effectiveness compared to conventional imaging techniques. By advancing preoperative imaging strategies, we can improve donor safety, optimize transplantation outcomes, and further integrate individualized medicine into clinical practice.

## Author Contributions

**Dominik Thomas Koch**: conceptualization, data curation, formal analysis, investigation, methodology, project administration, resources, software, supervision, validation, visualization, writing – original draft, writing – review and editing. **Felix Oliver Hofmann**: data curation, formal analysis, investigation, methodology, software, validation, visualization, writing – original draft, writing – review and editing. **Dimitrios Trompoukis**: data curation, investigation, methodology, software, validation, visualization, writing – review and editing. **Malte Schirren**: validation, writing – review and editing. **Severin Jacobi**: validation, writing – review and editing. **Tobias Seibt**: validation, writing – review and editing. **Stephan Kemmner**: validation, writing – review and editing. **Maximilian Scheifele**: writing – original draft, writing – review and editing. **Matthias Ilmer**: writing – review and editing. **Bernhard Renz**: supervision, writing – review and editing. **Jens Werner**: supervision, writing – review and editing. **Manfred Stangl**: supervision, writing – review and editing. **Markus Guba**: supervision, writing – review and editing. **Dionysios Koliogiannis**: conceptualization, data curation, formal analysis, investigation, methodology, project administration, resources, software, supervision, validation, visualization, writing – original draft, writing – review and editing.

## Ethics Statement

This study was approved by the Institute of Ethics, History and Theory of Medicine, Faculty of Medicine, LMU Munich, Germany (approval reference number 24‐0419).

## Conflicts of Interest

The authors declare no conflicts of interest.

## Data Availability

The data underlying this article will be shared on reasonable request to the corresponding author.
